# Association between Lifestyle Factors and Metabolic Syndrome among African Americans in the United States

**DOI:** 10.1155/2013/516475

**Published:** 2013-01-31

**Authors:** Chintan J. Bhanushali, Krishna Kumar, Anthony K. Wutoh, Spiridon Karavatas, Muhammad J. Habib, Marlon Daniel, Euni Lee

**Affiliations:** ^1^Department of Pharmaceutical Sciences, College of Pharmacy, Howard University, Washington, DC 20059, USA; ^2^Center for Minority Health Services Research, Department of Clinical and Administrative Pharmacy Sciences, College of Pharmacy, Howard University, Washington, DC 20059, USA; ^3^Department of Physical Therapy, College of Nursing and Allied Health Sciences, Howard University, Washington, DC 20059, USA

## Abstract

*Background*. Although there is a reported association between lifestyle factors and metabolic syndrome, very few studies have used national level data restricted to the African Americans (AAs) in the United States (US). *Methods*. A cross-sectional evaluation was conducted using the National Health and Nutrition Examination Survey from 1999 to 2006 including men and nonpregnant women of 20 years or older. Multiple logistic regression models were constructed to evaluate the association between lifestyle factors and metabolic syndrome. *Results*. AA women had a higher prevalence of metabolic syndrome (39.43%) than AA men (26.77%). After adjusting for sociodemographic factors, no significant association was found between metabolic syndrome and lifestyle factors including alcohol drinking, cigarette smoking, and physical activity. Age and marital status were significant predictors for metabolic syndrome. With increase in age, both AA men and AA women were more likely to have metabolic syndrome (AA men: OR_adj_ = 1.05, 95% CI 1.04–1.06, AA women: OR_adj_ = 1.06, 95% CI 1.04–1.07). Single AA women were less likely to have metabolic syndrome than married women (OR_adj_ = 0.66, 95% CI 0.43–0.99). *Conclusion*. Lifestyle factors had no significant association with metabolic syndrome but age and marital status were strong predictors for metabolic syndrome in AAs in the US.

## 1. Introduction

Metabolic syndrome refers to a cluster of known disorders that increase the risk for morbidity and mortality from cardiovascular disease (CVD) and type 2 diabetes [1, 2]. Risk for type 2 diabetes mellitus increases five- to ninefold with metabolic syndrome [1]. Metabolic syndrome is defined as the occurrence of 3 of any of the 5 following factors: obesity, elevated triglyceride (TG), low HDL-C, elevated blood pressure (BP), and elevated fasting glucose (FG) [3]. Lifestyle factors such as alcohol consumption, cigarette smoking, and physical activity have been reported to affect an individual's metabolic profile [4, 5]. A large population based study in the United States reported that mild to moderate alcohol consumption of alcohol was associated with a favorable influence on lipids, waist circumference, and fasting insulin in comparison to nondrinkers [4]. However, increased alcohol consumption has also been reported to be associated with hypertension [5]. Smoking has been associated with a negative lipid profile [6], hypertension [7, 8], and a higher risk of all forms of CVD [9]. Studies have also reported an inverse relationship between physical activity and certain components of metabolic syndrome such as waist circumference [10, 11], HDL-C [12, 13], and blood pressure [14, 15]. Although there has been limited number of studies reporting an association between lifestyle factors and metabolic syndrome in African American (AA) population [16–21], very few studies have used national level data.

The current study was designed to estimate gender specific prevalence of metabolic syndrome and to evaluate an association between lifestyle factors and metabolic syndrome in the AA population, using a nationally representative database, the National Health and Nutrition Examination Survey (NHANES).

## 2. Methods

### 2.1. Study Design and Data Source

This study utilized national level datasets available from the National Center for Health Statistics (NCHS) under the Center for Disease Control and Prevention [22]. The NCHS has conducted the NHANES from 1971 to 1994 focusing on different population groups or health topics [22]. The survey became a continuous program from 1999, examining a nationally representative sample of about 5,000 persons each year [22].

This study used datasets from 1999 to 2006, including all records from AA men and nonpregnant women who were 20 years or older who participated in the household interview and mobile examination center exam and attended the morning examination and fasted for at least 8.5 hours. Women who were reported as being pregnant were not included in this analysis. This exclusion criterion was established as waist circumference was used as an important indicator for determining the status of metabolic syndrome in our study, where pregnancy impacts waist circumference measurement.

### 2.2. Definition of Metabolic Syndrome and Lifestyle Variables

Metabolic syndrome was defined in this study as the occurrence of any three of the five components based on the criteria specified by ATP III [3]: (i) abdominal obesity, waist circumference ≥102 cm in men and ≥88 cm in women; (ii) elevated triglyceride (TG)—TG ≥150 mg/dL in both men and women or taking medication for dyslipidemia; (iii) low HDL-C—HDL-C value <40 mg/dL in men and <50 mg/dL in women; (iv) elevated blood pressure (BP)—systolic BP ≥135 mm Hg or diastolic BP ≥85 mm Hg, or taking medication for hypertension; (v) elevated fasting glucose (FG)—FG ≥100 mg/dL in both men and women, or taking medication for diabetes.

Lifestyle variables included alcohol consumption, cigarette smoking, and physical activity. Alcohol drinking status was created following guidelines from the US Department of Health and Human Services (HHS) [23] with three-level categories of *nondrinkers* (i.e., never drank alcohol during their lifetime or had quit alcohol drinking), *moderate drinker* (i.e., having no more than two drinks per day for men and no more than one drink per day for women, and heavy drinker using), and *heavy drinkers* (i.e., more than two drinks per day for men and more than one drink per day for women). Cigarette smoking was also categorized into a three-level variable as *nonsmokers* (i.e., those who never smoke cigarettes during their lifetime or who had quit cigarette smoking), *light smokers* (i.e., who smoked 1–10 cigarettes per day), and *heavy smokers* (i.e., who smoked more than 10 cigarettes per day). Physical activity was defined as a four-level categorical variable based on guidelines by HHS [24] including *inactivity* (i.e., no physical activity beyond baseline activities of daily living), *low activity* (i.e., less than 150 minutes (2 hours and 30 minutes) of moderate intensity physical activity a week, or the equivalent amount (75 minutes, or 1 hour and 15 minutes) of vigorous intensity activity), *medium activity* (i.e., moderate intensity activity of 150 minutes to 300 (5 hours), or 75 to 150 minutes of vigorous intensity physical activity per week), and *high activity* (i.e., more than the equivalent of 300 minutes of moderate intensity physical activity a week). Marital status, income, and educational levels were also included as socio-demographic variables using available data components from the database.

### 2.3. Statistical Analyses

The focus of the analyses was to estimate the prevalence of metabolic syndrome in AA men and women and to determine predictive factors for metabolic syndrome using a final analytic database that was constructed from 1999 to 2006, over the 4 cycles of the continuous NHANES: 1999-2000, 2001-2002, 2003-2004, and 2005-2006. National estimates were calculated using sampling weights available from the database using the survey functions in SAS, version 9.1.3 (SAS Institute, Cary, NC). All analyses were conducted using weighted data and variance estimates were calculated to account for the complex sampling methodology of NHANES. Relative standard errors (RSE) for all the weighted estimates were calculated and reported in our analysis. Gender specific logistic regression models were created to determine the association between lifestyle variables and metabolic syndrome while adjusting socio-demographic variables.

Bivariate and multivariate analyses were employed to examine the relationship between study variables and produced both unadjusted odds ratio (OR_unadj_) and adjusted OR (OR_adj_), respectively. The weighted estimates were calculated as either means or proportions, with the associated 95% confidence intervals (CI) and standard errors (SE).

## 3. Results

The characteristics of participants in the study are presented in Table 1. From the 1999–2006 NHANES, a total of 1,326 AA men and nonpregnant AA women (i.e., 654 AA men and 672 AA nonpregnant women) were identified as study population. Our study identified significant gender differences across socio-demographic and lifestyle factors as well as in the prevalence of metabolic syndrome along with its clinical components (Table 1). More specifically, a higher proportion of AA men reported being married and earning a higher income than AA women. A higher proportion of AA men were associated with alcohol drinking than AA women (65.85% versus 50.74%; *P* < 0.0001). The percentage of cigarette-smoking in AA women was lower than that in AA men (15.87% versus 28.58%; *P* < 0.0001). Metabolic syndrome was more prevalent in AA women than AA men (39.43% versus 26.77%; *P* < 0.0001). Findings also showed that there were gender differences among several components of metabolic syndrome (Table 1). For example, abdominal obesity was more prevalent in AA women (73.46%) than that in AA men (34.47%), but elevated triglyceride was more prevalent in AA men than AA women. HDL-C level was higher in AA women than AA men.

Predictive factors of metabolic syndrome were evaluated by bivariate and multivariate logistic regression analyses for AA men (Table 2) and AA women (Table 3). In AA men, age had a significant association with metabolic syndrome (OR_adj_ = 1.05, 95% CI 1.04–1.06) (Table 1). Although no significant association was found between lifestyle factors and metabolic syndrome for AA men from the adjusted model, bivariate analyses produced significant associations of metabolic syndrome with marital status (OR_unadj_ = 0.61, 95% CI 0.44–0.83), income (OR_unadj_ = 1.56, 95% CI 1.04–2.34), and heavy alcohol drinking (OR_unadj_ = 0.63, 95% CI 0.41–0.98) (Table 2).

Age was also a significant factor for metabolic syndrome in AA women (OR_adj_ = 1.06, 95% CI 1.04–1.07) (Table 3). Education, alcohol drinking, and physical activities were strong predictors for metabolic syndrome in bivariate model, but none of the lifestyle factors were significantly associated with metabolic syndrome and marital status was a significant factor from the multivariate model (OR_adj_ = 0.66, 95% CI 0.43–0.99) indicating that single AA women were less likely to have metabolic syndrome than married AA women (Table 3).

## 4. Discussion

Main findings of the study conclude that the prevalence of metabolic syndrome was higher in AA women (39.43%) than in men (26.77%). Higher prevalence of metabolic syndrome in AA women could be explained in part with the prevalence of abdominal obesity in AA women being twice as high as in AA men (73.46% versus 34.47%). Similar findings were documented in published literatures with higher prevalence of metabolic syndrome in AA women NHANES 2003–2006 [25] and a higher prevalence of obesity that are associated with high caloric food intake, little exercise, and dietary behavior influenced by cost, media, and cultural traditions [16–20, 26].

Our study showed that metabolic syndrome in AA men was more associated with income $45,000 or above as compared to men with income less than $20,000. Ogden and colleagues reported that AA men with higher income are more likely to be obese than those with low income [27]. Our findings on lower prevalence of metabolic syndrome in single AA women as compared to married women are in contrast to published reports that married people are more likely to engage in positive health behaviors than widowed, divorced, or single people [28, 29]. It is not clear how marital status contributes to metabolic syndrome, but a study by Troxel and colleagues reported marital quality as one of the important factors that mediates the association between marital status and metabolic syndrome [30].

Our findings showed no significant association between metabolic syndrome and lifestyle factors among AA men and AA women while literatures described alcohol drinking, cigarette smoking, and lack of physical activity as risk factors for metabolic syndrome [6, 31–37]. However, there are published studies also describing protective effects of smoking and alcohol on metabolic syndrome. For example, a U- or J-shaped relation between alcohol drinking and metabolic syndrome has been reported [38, 39]. A large population study using NHANES III by Freiberg and colleagues reported corroborating findings with our study that mild to moderate consumption of alcohol was associated with a favorable influence on lipids, waist circumference, and fasting insulin in comparison to current nondrinkers [4].

In regards to the impact of physical activities on metabolic syndrome, our study showed significant bivariate associations between physical activity and metabolic syndrome where medium or high activity level was a protective factor for metabolic syndrome in AA women. Ford and colleagues reported a similar finding on gender differences in relationship between physical activity and metabolic syndrome where physical inactivity was a predictive factor for metabolic syndrome in women but it was not a predictor in men [40]. More investigation might be needed to identify clearer relationships between lifestyle factors and metabolic syndrome among AA population.

One of the strengths of our study is that it provides unique information about metabolic syndrome in AA population. Very few studies have examined the association in AA and even fewer studies have utilized national representative databases with gender specific models. Although our findings did not find any associations between lifestyle factors like alcohol drinking, cigarette smoking, and physical activity and metabolic syndrome in both AA men and AA women, it highlighted the importance of early detection and intervention to reduce the risk of metabolic syndrome due to high blood pressure, elevated fasting glucose, and dyslipidemia among AA so that the progression of metabolic syndrome can be prevented at an early age.

There are several limitations to our study, primarily due to the data source being self-reports. The cross-sectional design of the study limits the ability in addressing causality and the present work only describes the association between lifestyle factors and metabolic syndrome.

## Figures and Tables

**Table 1:** Descriptive characteristics of the study population by gender using weighted estimates: from the NHANES 1999–2006.

Variables	Men	Women	*P* value
	Mean or percentage (SE)	Mean or percentage (SE)	
Age (yrs)	41.30 (0.58)	43.56 (0.64)	<0.05
Marital status (%)			
Married	52.00 (2.27)	39.28 (1.65)	<0.0001
Single	48.00 (2.27)	60.71 (1.65)	
Income (%)			
Less than $20,000	28.91 (1.65)	37.08 (2.55)	
$20,000–44,900	37.10 (1.94)	34.70 (2.16)	<0.05
$45,000 or above	33.99 (2.06)	28.22 (2.10)	
Education (%)			
Less than high school	31.70 (2.45)	24.09 (1.86)	
High school	25.39 (1.83)	23.78 (1.67)	<0.05
College or above	42.91 (2.10)	52.13 (1.83)	
Alcohol drinking (%)			
Nondrinkers	34.15 (2.34)	49.26 (1.85)	
Moderate drinkers	35.06 (1.73)	25.24 (1.33)	<0.0001
Heavy drinkers	30.79 (2.41)	25.50 (1.64)	
Cigarette smoking (%)			
Nonsmokers	71.41 (2.04)	84.13 (1.59)	
Light smokers	15.78 (1.53)	10.20 (1.30)	<0.0001
Heavy smokers	12.80 (1.84)	5.67 (0.97)	
Physical activity (%)			
Inactive	39.03 (2.01)	45.40 (1.99)	
Low activity	18.30 (1.56)	22.18 (1.75)	<0.05
Medium activity	11.26 (1.10)	12.83 (1.48)	
High activity	31.42 (1.91)	19.59 (1.76)	
Diastolic BP (mm Hg)	74.38 (0.61)	71.28 (0.54)	<0.05
Systolic BP (mm Hg)	128.28 (0.78)	126.58 (0.91)	0.15
Elevated BP (%)	49.71 (2.36)	50.06 (1.89)	0.53
Fasting glucose (mg/dL)	103.49 (1.33)	102.86 (1.29)	0.87
Triglyceride (mg/dL)	119.20 (4.24)	100.70 (2.29)	<0.05
HDL-C (mg/dL)	51.56 (0.55)	58.62 (0.59)	<0.0001
Abdominal obesity (%)	34.47 (2.16)	73.46 (1.65)	<0.0001
Waist circumference (cm)	97.02 (0.70)	99.68 (0.61)	<0.05
Metabolic syndrome (%)	26.77 (1.85)	39.43 (1.52)	<0.0001

**Table 2:** Predictive factors for metabolic syndrome among African American men using weighted estimates: from the NHANES 1999–2006*.

	OR_unadj_ (95% CI)	OR_adj_ (95% CI)
Age	**1.05 (1.04–1.06)**	**1.05 (1.04–1.06)**
Marital status		
Married	1.00	1.00
Single	**0.61 (0.44–0.83)**	0.85 (0.55–1.31)
Education		
Less than high school	1.00	1.00
High school	0.82 (0.51–1.31)	0.84 (0.48–1.47)
College graduate	0.81 (0.53–1.23)	0.75 (0.47–1.21)
Income		
<$20,000	1.00	1.00
$20,000–44,900	1.34 (0.88–2.02)	1.38 (0.88–2.16)
$45,000 or above	**1.56 (1.04–2.34)**	1.65 (0.92–2.97)
Alcohol drinking		
Nondrinkers	1.00	1.00
Moderate drinkers	0.96 (0.62–1.50)	1.13 (0.68–1.85)
Heavy drinkers	**0.63 (0.41–0.98)**	0.83 (0.48–1.45)
Cigarette smoking		
Nonsmoker	1.00	1.00
Light smoker	0.56 (0.31–1.01)	0.68 (0.36–1.27)
Heavy smoker	0.70 (0.40–1.24)	0.69 (0.39–1.22)
Physical activity		
Inactive	1.00	1.00
Low activity	0.86 (0.44–1.71)	1.10 (0.51–2.39)
Medium activity	0.70 (0.40–1.23)	0.97 (0.51–1.84)
High activity	0.62 (0.37–1.04)	0.94 (0.49–1.81)

*Any results with statistical significance with *P* < 0.05 are indicated in bold.

**Table 3:** Predictive factors for metabolic syndrome among African American women using weighted estimates: from the NHANES 1999–2006*.

	OR_unadj_ (95% CI)	OR_adj_ (95% CI)
Age	**1.05 (1.04–1.06)**	**1.06 (1.04–1.07)**
Marital status		
Married	1.00	1.00
Single	0.84 (0.60–1.17)	**0.66 (0.43–0.99)**
Education		
Less than high school	1.00	1.00
High school	0.68 (0.41–1.13)	1.08 (0.63–1.86)
College graduate	**0.56 (0.37–0.83)**	0.83 (0.50–1.39)
Income		
<$20,000	1.00	1.00
$20,000–44,900	1.06 (0.75–1.50)	1.03 (0.65–1.61)
$45,000 or above	0.79 (0.54–1.16)	0.68 (0.42–1.11)
Alcohol drinking		
Nondrinkers	1.00	1.00
Moderate drinkers	**0.74 (0.55–0.99)**	0.99 (0.68–1.43)
Heavy drinkers	**0.62 (0.44–0.88)**	1.12 (0.79–1.61)
Cigarette smoking		
Nonsmoker	1.00	1.00
Light smoker	0.85 (0.45–1.61)	0.74 (0.38–1.45)
Heavy smoker	1.51 (0.75–3.02)	1.18 (0.58–2.39)
Physical activity		
Inactive	1.00	1.00
Low activity	0.93 (0.61–1.42)	1.56 (0.93–2.62)
Medium activity	**0.54 (0.31–0.93)**	0.69 (0.31–1.56)
High activity	**0.60 (0.39–0.94)**	0.99 (0.60–1.64)

*Any results with statistical significance with *P* < 0.05 are indicated in bold.
